# Lantern-shaped screw loaded with autologous bone for treating osteonecrosis of the femoral head

**DOI:** 10.1186/s12891-018-2243-z

**Published:** 2018-09-05

**Authors:** Dasheng Lin, Lei Wang, Zhaoliang Yu, Deqing Luo, Xigui Zhang, Kejian Lian

**Affiliations:** 1 Orthopaedic Center of People’s Liberation Army, The Affiliated Southeast Hospital of Xiamen University, Zhangzhou, 363000 China; 2 Department of Surgery, Experimental Surgery and Regenerative Medicine, Ludwig-Maximilians-University (LMU), 80336 Munich, Germany; 3 Weigao Orthopaedic Device Co., Ltd, Weihai, 264200 China; 4 Double Engine Medical Material Co., Ltd, Xiamen, 361000 China

**Keywords:** Lantern-shaped screw, Autogenous bone graft, Core decompression, Osteonecrosis of the femoral head

## Abstract

**Background:**

Treatment for osteonecrosis of the femoral head (ONFH) in young individuals remains controversial. We developed a lantern-shaped screw, which was designed to provide mechanical support for the femoral head to prevent its collapse, for the treatment of ONFH. The purpose of this study was to investigate the efficacy and safety of the lantern-shaped screw loaded with autologous bone for the treatment of pre-collapse stages of ONFH.

**Methods:**

Thirty-two patients were randomly divided into two groups: the lantern-shaped screw group (core decompression and lantern-shaped screw loaded with autogenous bone) and the control group (core decompression and autogenous bone graft). During 36 months follow-up after surgery, treatment results in patients were assessed by X-ray and computed tomography (CT) scanning as well as functional recovery Harris hip score (HHS).

**Results:**

Successful clinical results were achieved in 15 of 16 hips (94%) in the lantern-shaped screw group compared with 10 of 16 hips (63%) in the control group (*p* = 0.0325). Successful radiological results were achieved in 14 of 16 hips (88%) in the lantern-shaped screw group compared with 8 of 16 hips (50%) in the control group (*P* = 0.0221).

**Conclusion:**

The lantern-shaped screw loaded with autologous bone for the treatment of pre-collapse stages of ONFH is effective and results in preventing progression of ONFH and reducing the risk of femoral head collapse.

**Trial registration:**

The trial registration number: ChiCTR-TRC-13004078 (retrospectively registered at 2013-11-28).

## Background

Hip-preserving surgery has been variable in treating osteonecrosis of the femoral head (ONFH), but there is a lack of consensus on the effectiveness of joint preserving procedures for ONFH. It is undesirable that hip replacement has been undertaken for the young individuals in whom hip prostheses have a limited survival. It is better to preserve the femoral head than replace it [1]. The exact mechanisms of femoral head collapse remain unclear. One hypothesis is based on the effects of shear stress at the boundary of necrotic and normal zones [2], and the other is in accordance with the grade of bone resorption at the boundary [3]. Karasuyama et al. [4] indicated that sclerotic differences at the boundary may play a crucial role in the pathomechanism of femoral head collapse. Core decompression with or without bone grafting are the most common technique for the early stages of ONFH [5–8]. Nevertheless, the current clinical results were not very satisfactory for patients in the early stages of ONFH performed with core decompression due to the lack of the sufficient structural support [9–11]. Various osteotomies including transtrochanteric rotational osteotomy and curved varus osteotomy have been presented well-known to treat ONFH [12]. Nevertheless, some studies have described various clinical results and risk factors for failure of the osteotomies, such as nonunion of the osteotomy and postoperative fracture of the femoral neck [13]. Porous tantalum implant procedure has been used for the management of the early stages of ONFH [14]. However, this procedure is neither entirely effective nor can it obtain predictable results [15–17]. It has been demonstrated that the implantation of a non-vascularized or vascularized fibula graft is a valuable treatment option for femoral head collapse prevention and hip function improvement in patients with pre-collapse osteonecrosis [18–21]. However, this technique may have certain drawbacks in that the implanted bone flaps would result in potential postoperative displacement. Improper post-operative weight-bearing onto the operated hip can also lead to the loosening of the implanted bone flaps, as well as poor bone regeneration and fusion [19, 22, 23].

In the actual practice, an ideal implant should be guaranteed to contact with the bone around the tunnel of the core decompression, as well as buttressing the subchondral bone of the femoral head. Collapse of femoral head will be less likely to occur when the implant contacted with the subchondral bone maximally [24]. Therefore, based on this principle, researchers have designed numerous devices for mechanical support of the femoral head, such as the super elastic cage implantation [25], the biomaterial-loaded allograft threaded cage [26], the umbrella-shaped memory alloy femoral head support device [27], PLGA/TCP scaffold [28], and cementation [29]. In our study, we developed a lantern-shaped screw, which was designed to provide the achievement of surface at surface support for the femoral head to prevent its collapse, for the treatment of ONFH. The purpose of this study was to investigate the efficacy and safety of a lantern-shaped screw loaded with autologous bone for the treatment of pre-collapse stages of ONFH.

## Methods

### Designing of the lantern-shaped screw

**Fig. 1:**
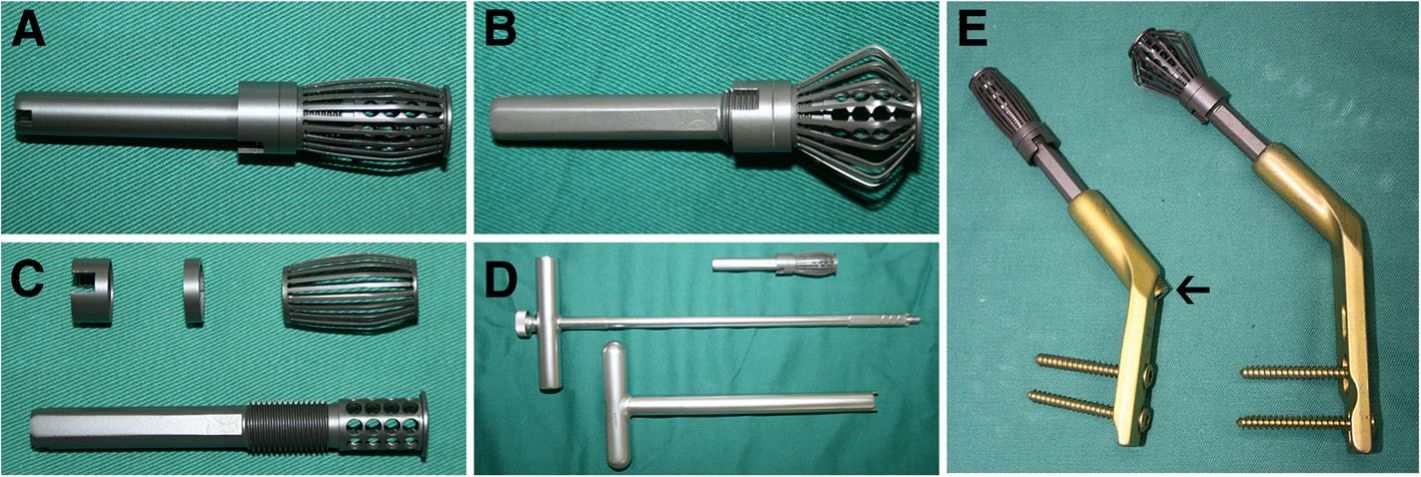
The lantern-shaped screw. **a** Photo of the folding lantern-shaped screw. **b** Photo of the unfolding lantern-shaped screw. **c** Photo of the structure and composition of the lantern-shaped screw. **d** Photo of the supporting equipment for the lantern-shaped screw. **e** Photo of the lantern-shaped screw and the angle plate with a locking tail cap (black arrow)

In collaboration with Weigao Orthopaedic Device Co., Ltd. (Weihai, China) and Double Engine Medical Material Co., Ltd. (Xiamen, China), we have designed a lantern-shaped screw, for which we have obtained a patent (patent number: CN103445851B). This screw is made of titanium alloy and cannulated, which is able to be full of the autogenous bone. The titanium mesh installed outside the lantern-shaped screw can be unfolded into the lantern shape to support the subchondral bone as much as possible. Furthermore, the tail cap of angle plate is a locking screw and can block the lantern-shaped screw back off (Fig. 1). Biomechanical tests of the lantern-shaped screws (*n* = 10) were done on the Dynacell, which is the truly dynamic load cell, designed from the outset for measuring dynamic loads (Instron, America). The results showed that the deformation behavior of the unfolded titanium mesh would not be varied until the strength of vertical compression was increased to 256.7 N (range 248–272 N). By acting the force of 500 N on the titanium mesh, the deformation was 1.9 mm (range 1.5–2.4 mm). These data demonstrated that the lantern-shaped screws have the sufficient mechanical strength to allow for the weight-bearing activities.

### Inclusion and exclusion criteria for this study

Patients were eligible for inclusion that aged 16 years to 50 years, suffered from ARCO stage II to III ONFH [19], and necrotic lesions occupying more than the medial 2/3 of the weight-bearing area [30]. Bone marrow oedema on magnetic resonance imaging (MRI) in the ONFH represents a secondary sign of subchondral fracture and thus indicates ARCO stage III [31]. The extent of the necrotic lesion within the femoral head was graded as Type A, B or C using the classification on the mid-coronal T1-weighted MRI scans. Type A lesions occupy less than 1/3 of the medial weight-bearing area or show no lesion on the mid-coronal MRI scan, Type B lesions occupy between 1/3 and 2/3 of the weight-bearing area, and Type C lesions occupy greater than 2/3 of the weight-bearing area [30]. Patients were excluded that had previous pathological fracture, or infection, or severe metabolic diseases, or cognitive impairment.

From January 2011 to December 2013, thirty-two patients with ONFH were eligible and enrolled into this study. There were twenty-five males and seven females that between 16 and 47 years of age. The duration from symptom onset to surgery treatment was 9 months to 38 months (Table 1). Patients who met the inclusion criteria were randomly divided into two groups: the lantern-shaped screw group (core decompression and lantern-shaped screw loaded with autogenous bone) and the control group (core decompression and autogenous bone graft). The randomization of the patients was done based on randomized numbers generated by sealed-envelope method.

**Table 1:** Baseline patient characteristics

Characteristic	Lantern-shaped screw group (*n* = 16)	Control group (*n* = 16)	*p* value
Mean age (range), yr.	31.5 ± 1.9 (16–47)	32.6 ± 1.7 (18–44)	0.6622
Female/ male, n	3/ 13	4/ 12	1.0000
Right/ left hip involved, n	9/ 7	10/ 6	0.7189
Body mass index (range), kg/cm^2^	24.9 ± 0.5 (21–27)	24.3 ± 0.5 (21–26)	0.3978
Etiology			0.4652
Nontraumatic, n	9	11	
Traumatic, n	7	5	
Duration of illness (range), mo	18.8 ± 2.0 (9–36)	20.5 ± 1.8 (11–38)	0.5379
ARCO stage (II/ III)	7/ 9	8/ 8	0.7232
Bone marrow edema of hip (III)	5	6	0.7097
Mean operation time (range), min	80.13 ± 3.3 (56–105)	71.4 ± 3.1 (45–90)	0.0621
Mean blood loss (range), mL	180.6 ± 14.9 (110–350)	158.8 ± 13.7 (90–280)	0.2892
Harris hip score			
Pre-operation (range)	60.3 ± 1.8 (48–72)	61.1 ± 2.0 (48–76)	0.7457
12 months (range)	86.3 ± 0.98 (81–94)	80.8 ± 1.5 (67–90)	0.0054
24 months (range)	85.6 ± 1.3 (70–92)	79.4 ± 1.7 (64–91)	0.0078
36 months (range)	85.2 ± 1.7 (68–95)	78.2 ± 2.2 (64–93)	0.0173
Successful clinical results (%)	15 (94)	10 (63)	0.0325
Successful radiological results (%)	14 (88)	8 (50)	0.0221

Detailed baseline patient characteristics are shown in Table 1. No significant differences were found in the baseline characteristics of the two groups, including patients’ age, gender, side of treated hip, body mass index, etiology, duration of illness, ARCO stage, and preoperative Harris hip score (HHS) [32].

### Surgical technique

All patients underwent surgery under spinal anesthesia or general anesthetic. After positioning of the patient in the supine position on an orthopedic traction table, the hip was exposed through a lateral approach, and a longitudinal incision was applied. With an incision of the subcutaneous tissue and the lateral fascia, the vastus lateralis muscle was separated by blunt dissection, and the proximal lateral femoral cortex was exposed. Core decompression was performed under C-arm fluoroscope using the standard technique [5, 6]. And then, the necrotic bone was removed locally.

**Fig. 2:**
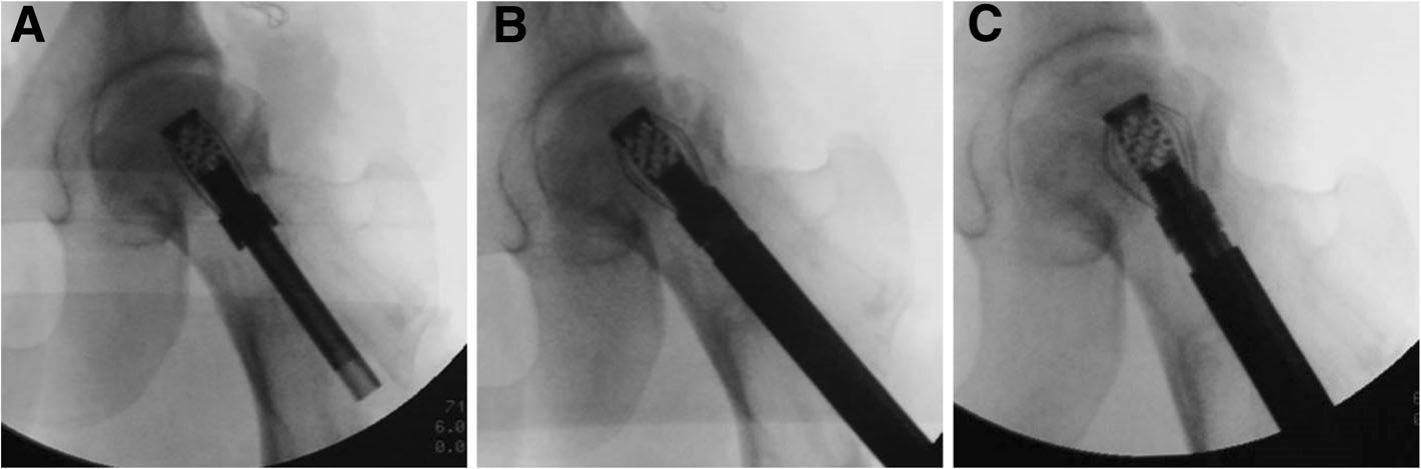
Intraoperative radiography for evaluation of surgical procedure. **a** Insertion of the lantern-shaped screw. **b** The lantern-shaped screw was being unfolded. **c** The lantern-shaped screw was the achievement of surface at surface support for the subchondral bone of femoral head

In the control group, the autogenous iliac-crest grafts were implanted into the region of the necrotic core through the bone channel. In the lantern-shaped screw group, the autogenous iliac-crest grafts were filled into the cannulated screw, and the rest of the autogenous bones were implanted into the region of the necrotic core. The bearing position of the lantern-shaped screw was a depth of approximate 7 mm beneath the articular cartilage surface. Then, the lantern-shaped screw was unfolded into the lantern shape using our designed reamer. Intraoperative C-arm fluoroscope confirmed the adequacy of positioning of the implant in the whole process (Fig. 2). Finally, the angle plate was fixed prior to closing the wound.

### Postoperative management and follow-up assessment

Antibiotic treatment was intravenously used within 24 h after surgery. The patients were rapidly mobilized and educated to be non-weight-bearing for 6 weeks. Partial weight-bearing with crutches or a walking aid was permitted for the following 6 weeks, and full-weight-bearing walking was allowed at 12 weeks postoperatively.

Clinically they were evaluated immediately after surgery and at 3, 6, 12, 24, and 36 months postoperatively. The HHS, which was acquired preoperatively and at 12, 24, and 36 months postoperatively, was used to evaluate the function of the hips. X-ray and computed tomography (CT) scans of the hips were experienced at the same follow-up times. The primary results of this study were clinical and radiological failure. Clinical failure was defined as HHS < 75 points or a requirement for subsequent hip surgery. New occurrence of collapse or increased collapse of greater than 2 mm on X-ray during follow-up was defined as radiological failure [11, 33, 34].

### Statistical analysis

SPSS 19.0 (SPSS Company, America) statistical software was used for statistical analysis. Baseline characteristics were assessed using descriptive statistics. The chi-square test was taken to compare nominal data. The *t*-test or Mann-Whitney *U* test was used to compare metric data. All statistical assessments were two-sided and evaluated at the 0.05 level of statistical significance.

## Results

**Fig. 3:**
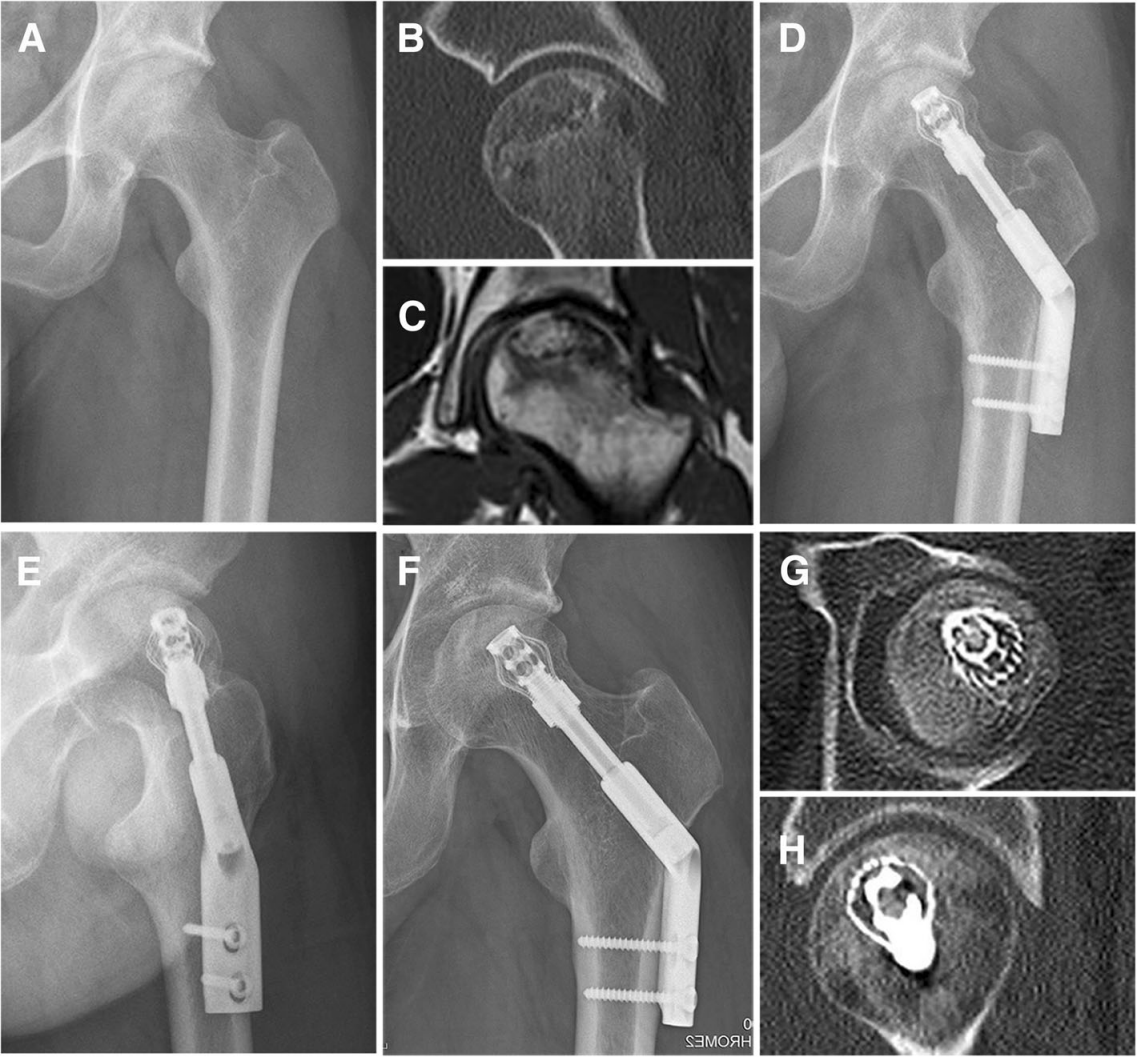
Representative radiographic images from both preoperative and postoperative taken at immediately after the lantern-shaped screw implantation and 36 months. **a-c** Preoperative X-ray, CT and sagittal T2-weighted magnetic resonance image showing ARCO stage III ONFH in a man aged 29 years. **d** and **e** X-ray anteroposterior view on the day of surgery. **f**-**h** X-ray and CT scans at 36 months after surgery showing union

**Fig. 4:**
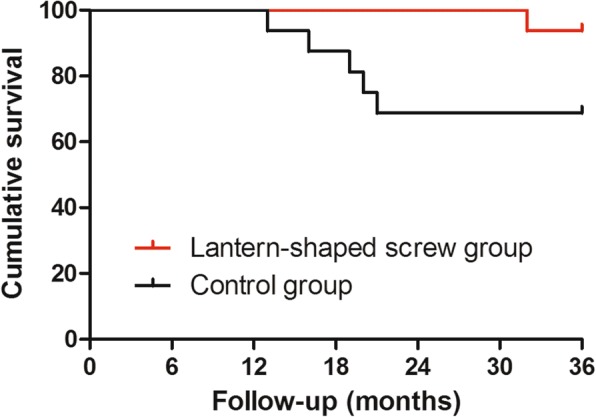
The figure shows survival with requirement for further hip surgery as the endpoint. The survival rate was different between the lantern-shaped screw group (94%) and the control group (69%) at 36 months (*P* = 0.0628)

The control group required less operation time and blood loss, but was not statistically different from the lantern-shaped screw group (Table 1). At 36 months follow-up, there was a significant difference between the preoperative and the last follow-up HHS in the lantern-shaped screw group (*p* < 0.0001) and control group (*P* < 0.0001). HHS was significantly improved in the lantern-shaped screw group when compared to the control group (*P* = 0.0173). The proportion of successful clinical results was significantly higher in the lantern-shaped screw group compared with the control group. Successful clinical results were achieved in 15 of 16 hips (94%) in the lantern-shaped screw group (Fig. 3). One hip (HHS was 68 points) required total hip replacement because of secondary degenerative arthritis at 32 months postoperatively, and was considered clinical failure. In the control group, successful clinical results were achieved in 10 of 16 hips (63%). Of the 6 hips that were clinical failures, three hips (HHS were 64, 67, and 68 points) underwent total hip replacement because of secondary degenerative arthritis 13, 19, and 20 months after surgery. Two (HHS were 64 and 71 points) underwent vascularized fibular grafting at 16 and 21 months after surgery and the remaining one (HHS was 73 points) had not undergone any further surgery at the last follow-up. The lantern-shaped screw group had a better radiological outcome than the control group (*P* = 0.0221). Successful radiological results were achieved in 14 of 16 hips (88%) in the lantern-shaped screw group compared with 8 of 16 hips (50%) in the control group (Table 1). The survival rates using requirement for further hip surgery as an endpoint were slight higher in the treatment group when compared with the control group (*P* = 0.0628; Fig. 4).

## Discussion

Our primary study utilizing our designed lantern-shaped screw for the treatment of pre-collapse stages of ONFH has generated promising effectiveness, with salvage of femoral head and improvement of the hip joint function at 36 months follow-up. A certain advantage of this screw is the achievement of surface at surface support between the weight-bearing area and upper surface of the lantern-shaped screw. Meanwhile, autogenous bone grafting promotes the bone regeneration and reconstruction.

Preservation of the collapse of the femoral head is the great predominant principle to treat patients with pre-collapse stages of ONFH despite of the unestablished pathogenesis. The rareness of bone repair microcirculation will cause osteonecrosis, primarily occurring in weight-bearing region of the femoral head [35]. However, many studies have suggested that the occurrence of the collapse is associated with the period of repair of necrotic area instead of period of ischemic necrosis [36, 37]. The repair can make the femoral head necrotic area construct again but it can also make bone structure alter or the mechanical properties decline in the process. The decline of mechanical properties is correlated directly with the collapse of the femoral head. The process of resorption of necrotic bone and replacement with new bone happen simultaneously [38]. Motomura et al. [2] who studied 30 femoral head specimens, found that in all of the femoral heads, collapse consistently involved a fracture at the lateral boundary of the necrotic lesion and that collapse began at the lateral boundary of the necrotic lesion and the size of the necrotic lesion seemed to contribute to its distribution. Consequently, in the clinical practice, preventing the collapse of subchondral bone has to be given sufficient biomechanical support and suitable circumstances to fulfill bone repair.

For many decades, surgical interventions for the hip-preserving have been controversial. Besides those techniques mentioned above, Papanagiotou et al. [39] reported a safe and effective method that non-vascularised fibular grafting with recombinant bone morphogenetic protein-7 for the management of ONFH in five of seven hips. They noted early consolidation of the non-vascularised fibular grafting and preventing collapse in pre-collapse stages. Yamasaki et al. [40] thought that it appeared to confer benefit in the repair of osteonecrosis and in the prevention of collapse by the transplantation of bone-marrow-derived mononuclear cells with a porous hydroxyapatite scaffold on early bone repair. Malizos et al. [41] introduced a technique using two or three 4.2 mm (or later 4.7 mm) tantalum pegs for the prevention of collapse of the necrotic lesion, finding that the estimated mean implant survival was 60 months. Chang et al. [42] reported that the poly (propylene fumarate) and calcium phosphate cement were combined to provide appropriate mechanical strength after core-decompressed femoral heads and offer the properties of osteoconductivity. Therefore, all the techniques are based on the mechanical support for the femoral head to prevent its collapse before the bone repair.

The goal of this procedure was to provide mechanical support of the articular surface while promoting bone healing and remodeling, and delay or avoid total hip replacement. The lantern-shaped screw is made of titanium with quite great intensity. Actually, the device is designed predominantly based on one of the characteristics of titanium, that is, it has great plasticity with a ductility property of up to 50–60%. The intensity allows the device to give sufficient support assistance and the plasticity is responsible for the transformation from the shape of cylinder into lantern. Furthermore, the tail cap of angle plate is locking screw and can block the lantern-shaped screw back off. The whole surgical procedure via the support device involved core decompression by decreasing intraosseous pressure to prevent the ischaemia, reconstruction of necrotic bone beneath the weight-bearing region, mechanical support of the articular surface via the surface at surface contact achieved by the lantern-shaped screw. Therefore, the lantern-shaped screw can be used in pre-collapse stages of ONFH.

### Limitations

This study has several limitations. Once the bone remodeling has occurred, the lantern-shaped screw has certain drawbacks in that it cannot be removed. Additionally, a small number of patients and short period of follow-up are the main limitations of this study. More studies with larger sample size and longer follow-up are required to accept the role of the lantern-shaped screw in ONFH.

## Conclusion

In summary, the biomechanical property and the bone remodeling regarding the lantern-shaped screw was verified, and the features of this device were also assessed. It is shown that the lantern-shaped screw loaded with autogenous bone may delay or avoid the progression of the collapse of the cartilage for the pre-collapse stages. It can not only provide appropriate circumstance to facilitate new bone remodeling but also strengthen the biomechanical structure for the weight-bearing region. We believe that the lantern-shaped screw could be used not only for autogenous bone graft but also for injection drugs, growth factor, mesenchymal stem cells and so on to be effective in early stages of ONFH. Therefore, we predict that the lantern-shaped screw could provide mechanical support and have considerable potential for medical application.

## Abbreviations

CT: Computed tomography
HHS: Harris hip score
MRI: Magnetic resonance imaging
ONFH: Osteonecrosis of the femoral head

## References

[1] Kose KC (2004). Femoral head resurfacing for the treatment of osteonecrosis in the young patient. Clin Orthop Relat Res.

[2] Motomura G, Yamamoto T, Yamaguchi R, Ikemura S, Nakashima Y, Mawatari T (2011). Morphological analysis of collapsed regions in osteonecrosis of the femoral head. J Bone Joint Surg Br..

[3] Li W, Sakai T, Nishii T, Nakamura N, Takao M, Yoshikawa H (2009). Distribution of TRAP-positive cells and expression of HIF-1alpha, VEGF, and FGF-2 in the reparative reaction in patients with osteonecrosis of the femoral head. J Orthop Res.

[4] Karasuyama K, Yamamoto T, Motomura G, Sonoda K, Kubo Y, Iwamoto Y (2015). The role of sclerotic changes in the starting mechanisms of collapse: a histomorphometric and FEM study on the femoral head of osteonecrosis. Bone.

[5] Hungerford DS (1988). Core decompression of the femoral head for osteonecrosis. J Bone Joint Surg Am.

[6] Warner JJ, Philip JH, Brodsky GL, Thornhill TS. Studies of nontraumatic osteonecrosis. The role of core decompression in the treatment of nontraumatic osteonecrosis of the femoral head. Clin Orthop Relat Res. 1987;(225):104–27. 3315373

[7] Marker DR, Seyler TM, Ulrich SD, Srivastava S, Mont MA (2008). Do modern techniques improve core decompression outcomes for hip osteonecrosis?. Clin Orthop Relat Res.

[8] Kerimaa P, Väänänen M, Ojala R, Hyvönen P, Lehenkari P, Tervonen O (2016). MRI-guidance in percutaneous core decompression of osteonecrosis of the femoral head. Eur Radiol.

[9] Zalavras CG, Lieberman JR (2014). Osteonecrosis of the femoral head: evaluation and treatment. J Am Acad Orthop Surg.

[10] Tabatabaee RM, Saberi S, Parvizi J, Mortazavi SM, Farzan M (2015). Combining concentrated autologous bone marrow stem cells injection with core decompression improves outcome for patients with early-stage osteonecrosis of the femoral head: a comparative study. J Arthroplast.

[11] Yin H, Yuan Z, Wang D (2016). Multiple drilling combined with simvastatin versus multiple drilling alone for the treatment of avascular osteonecrosis of the femoral head: 3-year follow-up study. BMC Musculoskelet Disord.

[12] Lee YK, Park CH, Ha YC, Kim DY, Lyu SH, Koo KH (2017). Comparison of surgical parameters and results between curved varus osteotomy and rotational osteotomy for osteonecrosis of the femoral head. Clin Orthop Surg.

[13] Ha YC, Kim HJ, Kim SY, Kim KC, Lee YK, Koo KH (2011). Effects of age and body mass index on the results of transtrochanteric rotational osteotomy for femoral head osteonecrosis: surgical technique. J Bone Joint Surg Am.

[14] Tsao AK, Roberson JR, Christie MJ, Dore DD, Heck DA, Robertson DD (2005). Biomechanical and clinical evaluations of a porous tantalum implant for the treatment of early-stage osteonecrosis. J Bone Joint Surg Am.

[15] Tanzer M, Bobyn JD, Krygier JJ, Karabasz D (2008). Histopathologic retrieval analysis of clinically failed porous tantalum osteonecrosis implants. J Bone Joint Surg Am.

[16] Varitimidis SE, Dimitroulias AP, Karachalios TS, Dailiana ZH, Malizos KN (2009). Outcome after tantalum rod implantation for treatment of femoral head osteonecrosis: 26 hips followed for an average of 3 years. Acta Orthop.

[17] Ma J, Sun W, Gao F, Guo W, Wang Y, Li Z (2016). Porous tantalum implant in treating osteonecrosis of the femoral head: still a viable option?. Sci Rep.

[18] Korompilias AV, Beris AE, Lykissas MG, Kostas-Agnantis IP, Soucacos PN (2011). Femoral head osteonecrosis: why choose free vascularized fibula grafting. Microsurgery.

[19] Zhao D, Huang S, Lu F, Wang B, Yang L, Qin L (2016). Vascularized bone grafting fixed by biodegradable magnesium screw for treating osteonecrosis of the femoral head. Biomaterials.

[20] Korompilias AV, Lykissas MG, Beris AE, Urbaniak JR, Soucacos PN (2009). Vascularised fibular graft in the management of femoral head osteonecrosis: twenty years later. J Bone Joint Surg Br..

[21] Zhang CQ, Sun Y, Chen SB, Jin DX, Sheng JG, Cheng XG (2011). Free vascularised fibular graft for post-traumatic osteonecrosis of the femoral head in teenage patients. J Bone Joint Surg Br.

[22] Malizos KN, Quarles LD, Dailiana ZH, Rizk WS, Seaber AV, Urbaniak JR (2004). Analysis of failures after vascularized fibular grafting in femoral head necrosis. Orthop Clin North Am.

[23] Dailiana ZH, Toth AP, Gunneson E, Berend KR, Urbaniak JR (2007). Free vascularized fibular grafting following failed core decompression for femoral head osteonecrosis. J Arthroplast.

[24] Civinini R, De Biase P, Carulli C, Matassi F, Nistri L, Capanna R (2012). The use of an injectable calcium sulphate/calcium phosphate bioceramic in the treatment of osteonecrosis of the femoral head. Int Orthop.

[25] Wang Y, Chai W, Wang ZG, Zhou YG, Zhang GQ, Chen JY (2009). Superelastic cage implantation: a new technique for treating osteonecrosis of the femoral head with mid-term follow-ups. J Arthroplasty.

[26] Yang S, Wu X, Xu W, Ye S, Liu X, Liu X (2010). Structural augmentation with biomaterial-loaded allograft threaded cage for the treatment of femoral head osteonecrosis. J Arthroplast.

[27] Yu X, Jiang W, Pan Q, Wu T, Zhang Y, Zhou Z (2013). Umbrella-shaped, memory alloy femoral head support device for treatment of avascular osteonecrosis of the femoral head. Int Orthop.

[28] Qin L, Yao D, Zheng L, Liu WC, Liu Z, Lei M (2015). Phytomolecule icaritin incorporated PLGA/TCP scaffold for steroid-associated osteonecrosis: proof-of-concept for prevention of hip joint collapse in bipedal emus and mechanistic study in quadrupedal rabbits. Biomaterials.

[29] Wood ML, McDowell CM, Kelley SS (2003). Cementation for femoral head osteonecrosis: a preliminary clinic study. Clin Orthop Relat Res.

[30] Nishii T, Sugano N, Ohzono K, Sakai T, Haraguchi K, Yoshikawa H (2002). Progression and cessation of collapse in osteonecrosis of the femoral head. Clin Orthop Relat Res.

[31] Meier R, Kraus TM, Schaeffeler C, Torka S, Schlitter AM, Specht K (2014). Bone marrow oedema on MR imaging indicates ARCO stage 3 disease in patients with AVN of the femoral head. Eur Radiol.

[32] Harris WH (1969). Traumatic arthritis of the hip after dislocation and acetabular fractures: treatment by mold arthroplasty. An end-result study using a new method of result evaluation. J Bone Joint Surg Am.

[33] Lee MS, Hsieh PH, Chang YH, Chan YS, Agrawal S, Ueng SW (2008). Elevated intraosseous pressure in the intertrochanteric region is associated with poorer results in osteonecrosis of the femoral head treated by multiple drilling. J Bone Joint Surg Br..

[34] Kang P, Pei F, Shen B, Zhou Z, Yang J (2012). Are the results of multiple drilling and alendronate for osteonecrosis of the femoral head better than those of multiple drilling? A pilot study. Joint Bone Spine.

[35] Mont MA, Hungerford DS (1995). Non-traumatic avascular necrosis of the femoral head. J Bone Joint Surg Am.

[36] Brown TD, Way ME, Ferguson AB Jr. Mechanical characteristics of bone in femoral capital aseptic necrosis. Clin Orthop Relat Res. 1981;(156):240–7. 7226659

[37] Ueo T, Tsutsumi S, Yamamuro T, Okumura H, Shimizu A, Nakamura T (1985). Biomechanical aspects of the development of aseptic necrosis of the femoral head. Arch Orthop Trauma Surg.

[38] Kim HJ (2004). Hyperbaric oxygen therapy as a treatment for stage-I avascular necrosis of the femoral head. J Bone Joint Surg Br..

[39] Papanagiotou M, Malizos KN, Vlychou M, Dailiana ZH (2014). Autologous (non-vascularised) fibular grafting with recombinant bone morphogenetic protein-7 for the treatment of femoral head osteonecrosis: preliminary report. Bone Joint J.

[40] Yamasaki T, Yasunaga Y, Ishikawa M, Hamaki T, Ochi M (2010). Bone-marrow-derived mononuclear cells with a porous hydroxyapatite scaffold for the treatment of osteonecrosis of the femoral head: a preliminary study. J Bone Joint Surg Br..

[41] Malizos KN, Papasoulis E, Dailiana ZH, Papatheodorou LK, Varitimidis SE (2012). Early results of a novel technique using multiple small tantalum pegs for the treatment of osteonecrosis of the femoral head: a case series involving 26 hips. J Bone Joint Surg Br..

[42] Chang CH, Liao TC, Hsu YM, Fang HW, Chen CC, Lin FH (2010). A poly (propylene fumarate)--calcium phosphate based angiogenic injectable bone cement for femoral head osteonecrosis. Biomaterials.

